# Hepatic steatosis index as an independent predictor of hypertension in patients with type 2 diabetes: a cross-sectional study

**DOI:** 10.3389/fmed.2025.1697412

**Published:** 2025-11-06

**Authors:** Xuan Ma, Qingyi Zhou, Chen Ma, Jie Sheng, Xinghe Jiang, Guanqi Gao, Baolan Ji

**Affiliations:** 1 School of Clinical Medicine, Shandong Second Medical University, Weifang, Shandong, China; 2 Department of Endocrinology, Linyi People's Hospital Affiliated to Shandong Second Medical University, Linyi, Shandong, China; 3 Second Affiliated Hospital, Bengbu Medical University, Bengbu, Anhui, China; 4 School of Chinese Medicine, Beijing University of Chinese Medicine, Beijing, China

**Keywords:** type 2 diabetes mellitus, hypertension, hepatic steatosis index, metabolic dysfunction-associated steatotic liver disease, metabolic dysfunction

## Abstract

**Background:**

Hypertension is a common comorbidity in type 2 diabetes mellitus (T2DM) and increases cardiovascular risk. Hepatic steatosis, a hallmark of metabolic dysfunction frequently observed in T2DM, may contribute to elevated blood pressure. The hepatic steatosis index (HSI) is a simple, non-invasive marker of liver fat, but its predictive value for hypertension in T2DM patients remains unclear.

**Methods:**

This cross-sectional study retrospectively included 1,744 hospitalized T2DM patients at Linyi People’s Hospital from 2020 to 2023. Demographic, anthropometric, and laboratory data were collected, and HSI was calculated. Patients were classified as hypertensive (*n* = 604) or non-hypertensive (*n* = 1,140) and further stratified by HSI quartiles (Q1–Q4). Univariate and multivariate logistic regression analyses were performed to assess the association between HSI and hypertension.

**Results:**

Patients with hypertension had significantly higher HSI levels than those without (*p* < 0.05). Hypertension prevalence increased progressively across HSI quartiles, with the highest in Q4 (*p* < 0.01). In multivariate analysis, after adjustment for potential confounders, HSI remained independently associated with hypertension (OR = 1.054; 95% CI: 1.025–1.085; *p* < 0.001).

**Conclusion:**

HSI is independently associated with hypertension in T2DM and may serve as a practical tool for risk stratification. These findings underscore the link between hepatic steatosis and cardiovascular risk, and further studies are warranted to confirm causality and clinical utility.

## Introduction

Type 2 diabetes mellitus (T2DM) is a prevalent chronic metabolic disorder with rising global incidence (1). Hypertension is one of the most common comorbidities in T2DM, affecting over half of patients, and substantially increases the risk of cardiovascular complications and diabetes-related morbidity (2, 3). Early identification of factors associated with hypertension is therefore crucial for risk stratification and timely intervention in T2DM populations.

Metabolic dysfunction, particularly hepatic fat accumulation, plays a central role in the development of hypertension in T2DM (4). The liver, as a key organ in metabolic regulation, contributes to blood pressure homeostasis through mechanisms such as insulin resistance, systemic inflammation, and endothelial dysfunction (5). Metabolic dysfunction-associated steatotic liver disease (MASLD), a frequent manifestation of metabolic dysregulation in T2DM, has been implicated in elevated blood pressure and increased cardiovascular risk (6–9). However, traditional assessment methods for hepatic steatosis, such as imaging and liver biopsy, are limited by cost, invasiveness, and practical difficulties for large-scale screening (10).

The hepatic steatosis index (HSI) is a non-invasive and easily obtainable surrogate marker for liver fat. It is calculated from routine clinical parameters, including body mass index (BMI), sex, and the ALT/AST ratio, providing a more accessible tool for identifying individuals at risk of metabolic dysfunction (11). Previous studies have linked HSI to insulin resistance, dyslipidemia, and metabolic syndrome, suggesting a potential relationship with hypertension (12, 13). Despite these findings, comprehensive evidence on the role of HSI as a predictor of hypertension in patients with T2DM.

In this study, we aimed to investigate the association between HSI and hypertension in patients with T2DM, focusing on how HSI influences blood pressure regulation through metabolic dysfunction, insulin resistance, and hepatic fat accumulation. Statistical analysis revealed the strength and nature of this association and explored the potential clinical utility of HSI in predicting hypertension risk, thereby supporting individualized risk stratification and precision management for T2DM patients.

## Methods

### Study participants

This was a retrospective cross-sectional study in which data from patients with T2DM admitted to Linyi People’s Hospital between January 2020 and March 2023 were collected to explore the association between HSI and hypertension.

Inclusion criteria were adults (≥18 years) with a confirmed diagnosis of T2DM according to the 1999 World Health Organization (WHO) criteria.

Exclusion criteria were as follows (1) type 1 diabetes or acute diabetic complications; (2) significant hepatic impairment (defined as ALT or AST levels exceeding twice the upper normal limit) or renal dysfunction (eGFR < 90 mL/min/1.73 m^2^); (3) any documented secondary cause of hypertension; and (4) missing key laboratory data required for the calculation of the HSI.

**Figure 1:**
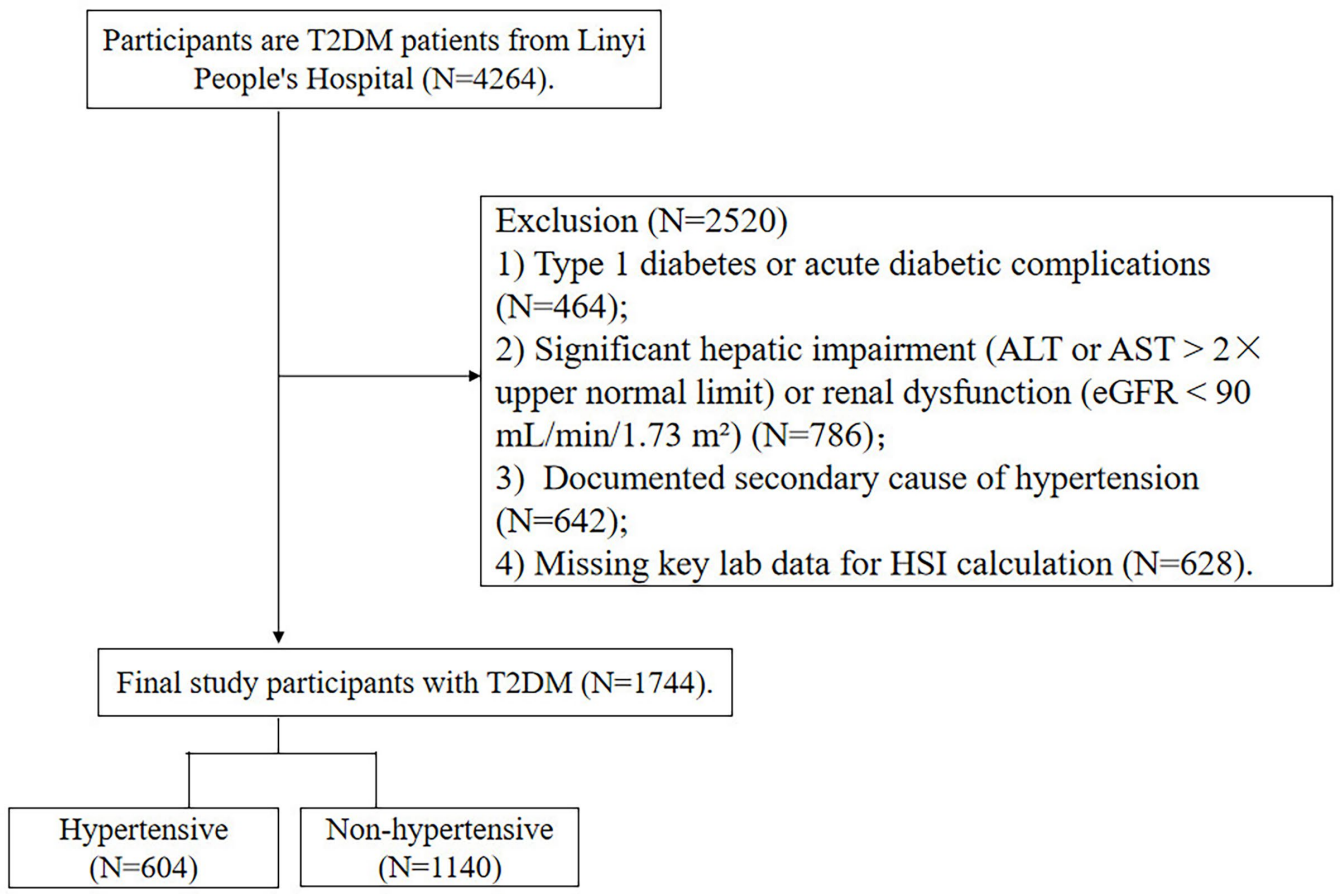
Flowchart of participant selection. From 4,264 hospitalized T2DM patients screened, exclusions were applied for type 1 diabetes or acute diabetic complications, hepatic or renal dysfunction, secondary causes of hypertension, and missing key laboratory data, yielding a final sample of 1,744 participants (604 hypertensive; 1,140 non-hypertensive).

After applying these criteria, a total of 1,744 patients (1,042 females and 702 males) were included in the final analysis (Figure 1).

### Anthropometric and biochemical measurements

Anthropometric and biochemical measurements were obtained following standardized procedures. Demographic and clinical information was recorded, including age, sex, duration of diabetes, height, weight, smoking status, and alcohol consumption. Visceral fat area (VFA) and subcutaneous fat area (SFA) were measured using a bioelectrical impedance analyzer (HDS-2000, Omron, Kyoto, Japan).

Fasting venous blood samples were collected the following morning after at least 8 h of overnight fasting. Biochemical analyses were performed using a fully automated chemistry analyzer (Cobas c702, Roche Diagnostics, Germany) and included total cholesterol (TC), triglycerides (TG), high-density lipoprotein cholesterol (HDL-c), low-density lipoprotein cholesterol (LDL-c), alanine aminotransferase (ALT), aspartate aminotransferase (AST), gamma-glutamyl transferase (GGT), serum creatinine (Scr), uric acid (UA), fasting plasma glucose (FPG), glycated hemoglobin (HbA1c, measured by high-performance liquid chromatography), and hemoglobin (Hb). Urinary albumin-to-creatinine ratio (UACR) was determined using an automated analyzer (Beckman Coulter AU5821).

Blood pressure was measured on the non-dominant arm using an automated electronic sphygmomanometer (HDS-2000, Omron, Kyoto, Japan) after participants had been seated quietly for at least five minutes. Two consecutive readings were taken at two-minute intervals, and the average value was used for analysis. Hypertension was defined as systolic blood pressure (SBP) ≥ 140 mmHg, diastolic blood pressure (DBP) ≥ 90 mmHg, or a previous diagnosis of hypertension with ongoing use of antihypertensive treatment.

### Rationale for choosing HSI

HSI was chosen for its simplicity, non-invasive nature, and reliance on routine clinical parameters such as BMI, ALT, and AST, which are commonly measured in clinical practice. Unlike imaging-based methods, HSI does not require specialized equipment or advanced technology, making it more accessible and cost-effective, particularly for large-scale studies and routine monitoring of T2DM patients.

### Parameter calculations

1. BMI = weight (kg)/height (m)^2^.
2. eGFR = 175 × Scr (mg/dL)^−1.234^ × age^−0.179^ × (0.79, if female) (14).

The Modification of Diet in Renal Disease (MDRD) equation was used to estimate eGFR, as it has been validated in diabetic and CKD populations and is routinely applied in our institution for kidney function assessment.

HSI was calculated as 8 × (ALT/AST ratio) + BMI (+2 if female, +2 if diabetes) (11), according to the formula proposed by Lee et al. (11). ALT and AST were measured in U/L using a fully automated chemistry analyzer (Cobas c702, Roche Diagnostics, Germany), and BMI was calculated as described above.

### Statistical analysis

All statistical analyses were performed using SPSS version 26.0 (IBM Corp., Chicago, IL, United States). Continuous variables were expressed as mean ± standard deviation (SD) or median (interquartile range, IQR) as appropriate. Group comparisons were conducted using the independent-samples *t*-test, Mann–Whitney U test, one-way ANOVA, or Chi-square test, as appropriate.

Both univariate and multivariate logistic regression analyses were performed to identify factors associated with hypertension. Variables that were significant in the univariate analysis were entered into the multivariate model to adjust for potential confounding factors. A two-tailed *p*-value <0.05 was considered statistically significant.

## Results

### Clinical characteristics of the study subjects

Table 1 displays the clinical and biochemical characteristics of the study population. Patients were categorized into a non-hypertensive group (*n* = 1,140) and a hypertensive group (*n* = 604). Compared with non-hypertensive patients, those with hypertension were older, had a longer diabetes duration, and exhibited higher BMI, SBP, DBP, VFA, and SFA (all *p* < 0.001). In contrast, FPG and HbA1c levels were significantly lower in the hypertensive group (*p* = 0.001 and *p* < 0.001, respectively). With respect to lipid profiles and liver and kidney function, hypertensive patients demonstrated higher LDL-c, AST, GGT, UA, Scr, UACR, and HSI (all *p* < 0.05), along with reduced eGFR (*p* < 0.001). No significant differences were found in sex distribution, smoking and alcohol consumption, TC, HDL-c, or ALT between the two groups (all *p* > 0.05).

**Table 1:** Comparison of clinical and biochemical characteristics between non-hypertension and hypertension groups.

Variables	Non-hypertension	Hypertension	*P*
Number	1,140	604	
Sex (females, *n*, %)	672 (58.9%)	371 (61.4%)	0.316
Age (years)	55.00 ± 12.47	63.02 ± 10.30	<0.001
Diabetes duration (years)	7 (2,11)	10 (4,16)	<0.001
VFA (cm^2^)	85.00 (60.00, 110.00)	102.00 (75.25, 131.00)	<0.001
SFA (cm^2^)	172.5 (130.00, 218.00)	199.00 (160.00, 241.00)	<0.001
Smoking (*n*, %)	181 (15.9%)	97 (16.1%)	0.927
Alcohol consumption (*n*, %)	157 (13.8%)	91 (15.1%)	0.466
BMI (kg/m^2^)	25.02 ± 3.59	26.35 ± 3.55	<0.001
SBP (mmHg)	124.51 ± 15.83	140.15 ± 20.41	<0.001
DBP (mmHg)	78.72 ± 10.34	83.02 ± 13.08	<0.001
Hb (g/L)	140.92 ± 17.85	138.29 ± 18.34	0.004
TC (mmol/L)	4.87 ± 1.32	4.74 ± 1.38	0.061
LDL-c (mmol/L)	2.92 ± 1.07	3.08 ± 1.00	0.002
TG (mmol/L)	1.37 (0.95, 2.02)	1.47 (1.05, 2.16)	0.010
HDL-c (mmol/L)	1.18 ± 0.32	1.19 ± 0.38	0.652
FPG (mmol/L)	9.27 ± 3.99	8.65 ± 3.34	0.001
HbA1c (%)	9.59 ± 2.31	8.91 ± 2.08	<0.001
ALT (U/L)	17.30 (12.93, 26.18)	18.65 (13.60, 25.78)	0.074
AST (U/L)	17.20 (13.90, 22.60)	17.85 (15.13, 21.90)	0.017
GGT (U/L)	21.00 (14.00, 31.00)	22.00 (16.00, 33.00)	0.006
UA (μmolL)	280.99 ± 94.82	293.31 ± 94.48	0.010
Scr (μmol/L)	62.13 ± 20.20	69.51 ± 27.35	<0.001
eGFR (mL/min/1.73 m^2^)	125.86 ± 33.93	110.52 ± 36.45	<0.001
UACR (mg/g)	9.30 (5.20, 25.90)	15.45 (7.30, 74.48)	<0.001
HSI	36.68 (33.40, 40.00)	37.91 (35.25, 41.15)	<0.001

VFA, visceral fat area; SFA, subcutaneous fat area; BMI, body mass index; SBP, systolic blood pressure; DBP, diastolic blood pressure; Hb, hemoglobin; TC, total cholesterol; LDL-c, low-density lipoprotein cholesterol; TG, triglyceride; HDL-c, high-density lipoprotein cholesterol; FPG, fasting plasma glucose; HbA1c, glycated hemoglobin; ALT, alanine aminotransferase; AST, aspartate aminotransferase; GGT, γ-glutamyl transpeptidase; UA, uric acid; Scr, serum creatinine; eGFR, estimated glomerular filtration rate; UACR, urinary albumin-to-creatinine ratio; HSI, hepatic steatosis index. Data were presented as mean ± SD for normally distributed variables, and as median (interquartile range) for non-normally distributed variables. Independent-Samples *t* test and Mann–Whitney U test were applied for comparisons of normally and non-normally distributed continuous variables between the Non-hypertension and Hypertension groups, respectively. Categorical variables were expressed as *n* (%), and were compared using the chi-square test. Statistical significance was defined as a two-tailed *p* value <0.05.

Table 2 summarizes the clinical and biochemical variables stratified by HSI quartiles. As HSI increased, patients exhibited progressively higher VFA, SFA, BMI, SBP, DBP, Hb, TG, ALT, AST, GGT, and UA, whereas HDL-c levels decreased (all *p* < 0.001). Detailed descriptive statistics (mean ± SD for normally distributed variables or median [IQR] for skewed variables), along with corresponding *p* values, are presented in Tables 1, 2 to illustrate the distribution and significance of these variables across HSI quartiles. Age and diabetes duration showed an inverse association with HSI (both *p* < 0.001). FPG and HbA1c levels increased modestly across quartiles (*p* = 0.005 and *p* = 0.045, respectively). In contrast, no significant differences were observed for sex distribution, smoking, alcohol consumption, TC, LDL-c, and Scr. eGFR increased slightly with higher HSI (*p* = 0.030), whereas UACR levels declined (*p* = 0.001). Notably, the prevalence of hypertension rose steadily across HSI quartiles (23.6, 34.2, 39.1, and 41.6%, respectively; *p* < 0.001).

**Table 2:** Comparison of variables according to the quartiles of the HSI.

Variables	Q1 (23.30, 33.95)	Q2 (33.95, 37.16)	Q3 (37.16, 40.44)	Q4 (40.44, 67.92)	*P*
Females	256 (58.7%)	276 (63.3%)	252 (57.9%)	259 (59.3%)	0.374
Age	58.87 ± 12.86	59.41 ± 10.85	58.63 ± 11.09	54.21 ± 13.75	<0.001
Diabetes duration	9.50 (3.00, 15.00)	8.00 (4.00, 13.00)	10.00 (4.00, 13.00)	5.00 (2.00, 10.00)	<0.001
VFA	60.00 (36.00, 82.00)	81.00 (62.00, 102.75)	98.00 (79.00, 122.00)	121.00 (96.00, 150.50)	<0.001
SFA	124.00 (92.75, 161.00)	166.00 (138.00, 199.00)	200.00 (164.00, 230.00)	242.00 (200.50, 288.00)	<0.001
Smoking	66 (15.1%)	56 (12.8%)	72 (16.6%)	84 (19.2%)	0.074
Alcohol consumption	62 (14.2%)	53 (12.2%)	62 (14.3%)	71 (16.2%)	0.393
BMI	21.79 ± 2.13	24.51 ± 1.94	26.28 ± 2.03	29.32 ± 3.23	<0.001
SBP	125.10 ± 19.04	129.91 ± 18.75	131.45 ± 19.14	133.28 ± 18.39	<0.001
DBP	77.16 ± 11.31	79.15 ± 10.85	80.19 ± 10.46	84.32 ± 12.30	<0.001
Hb	133.98 ± 18.88	139.30 ± 16.75	141.38 ± 17.75	145.43 ± 16.94	<0.001
TC	4.74 ± 1.28	4.83 ± 1.35	4.80 ± 1.35	4.94 ± 1.36	0.139
LDL-c	2.97 ± 0.96	2.99 ± 1.09	3.01 ± 0.98	3.11 ± 1.07	0.181
TG	1.05 (0.77, 1.54)	1.33 (1.02, 1.94)	1.51 (1.12, 2.14)	1.75 (1.22, 2.72)	<0.001
HDL-c	1.28 ± 0.35	1.22 ± 0.40	1.15 ± 0.32	1.09 ± 0.28	<0.001
FPG	8.70 ± 4.38	8.93 ± 3.43	9.02 ± 3.37	9.57 ± 3.59	0.005
HbA1c	9.58 ± 2.58	9.22 ± 2.19	9.21 ± 2.11	9.41 ± 2.11	0.045
ALT	13.20 (10.30, 17.38)	16.20 (12.80, 21.55)	20.00 (14.80, 27.10)	26.20 (18.50, 42.05)	<0.001
AST	16.70 (13.63, 21.08)	17.10 (14.20, 20.95)	17.70 (14.40, 21.90)	18.50 (15.05, 26.90)	<0.001
GGT	17.00 (12.00, 24.00)	19.60 (14.00, 28.00)	23.00 (16.70, 33.00)	28.00 (20.00, 41.05)	<0.001
UA	272.26 ± 102.11	277.52 ± 92.32	283.85 ± 85.54	307.43 ± 95.19	<0.001
Scr	66.49 ± 23.65	65.61 ± 25.51	63.78 ± 20.44	62.85 ± 22.77	0.080
eGFR	116.73 ± 32.62	118.12 ± 35.86	121.49 ± 35.79	125.84 ± 37.28	0.030
UACR	12.95 (6.10, 60.30)	11.10 (5.90, 35.65)	10.40 (5.90, 28.10)	10.40 (5.60, 30.75)	0.001
Hypertension	103 (23.6%)	149 (34.2%)	170 (39.1%)	182 (41.6%)	<0.001

HSI, hepatic steatosis index; VFA, visceral fat area; SFA, subcutaneous fat area; BMI, body mass index; SBP, systolic blood pressure; DBP, diastolic blood pressure; Hb, hemoglobin; TC, total cholesterol; LDL-c, low-density lipoprotein cholesterol; TG, triglyceride; HDL-c, high-density lipoprotein cholesterol; HbA1c, glycated hemoglobin; ALT, alanine aminotransferase; AST, aspartate aminotransferase; GGT, γ-glutamyl transpeptidase; UA, uric acid; Scr, serum creatinine; eGFR, estimated glomerular filtration rate; UACR, urinary albumin-to-creatinine ratio. Data were presented as mean ± SD for normally distributed variables and as median (interquartile range) for skewed variables. For comparisons among HSI quartiles, one-way analysis of variance (ANOVA) was applied for normally distributed continuous variables, while the Kruskal–Wallis test was used for non-normally distributed continuous variables. Categorical variables were expressed as *n* (%), and were compared using the chi-square test. Statistical significance was defined as a two-tailed *p* value <0.05.

### Univariate analysis

**Figure 2:**
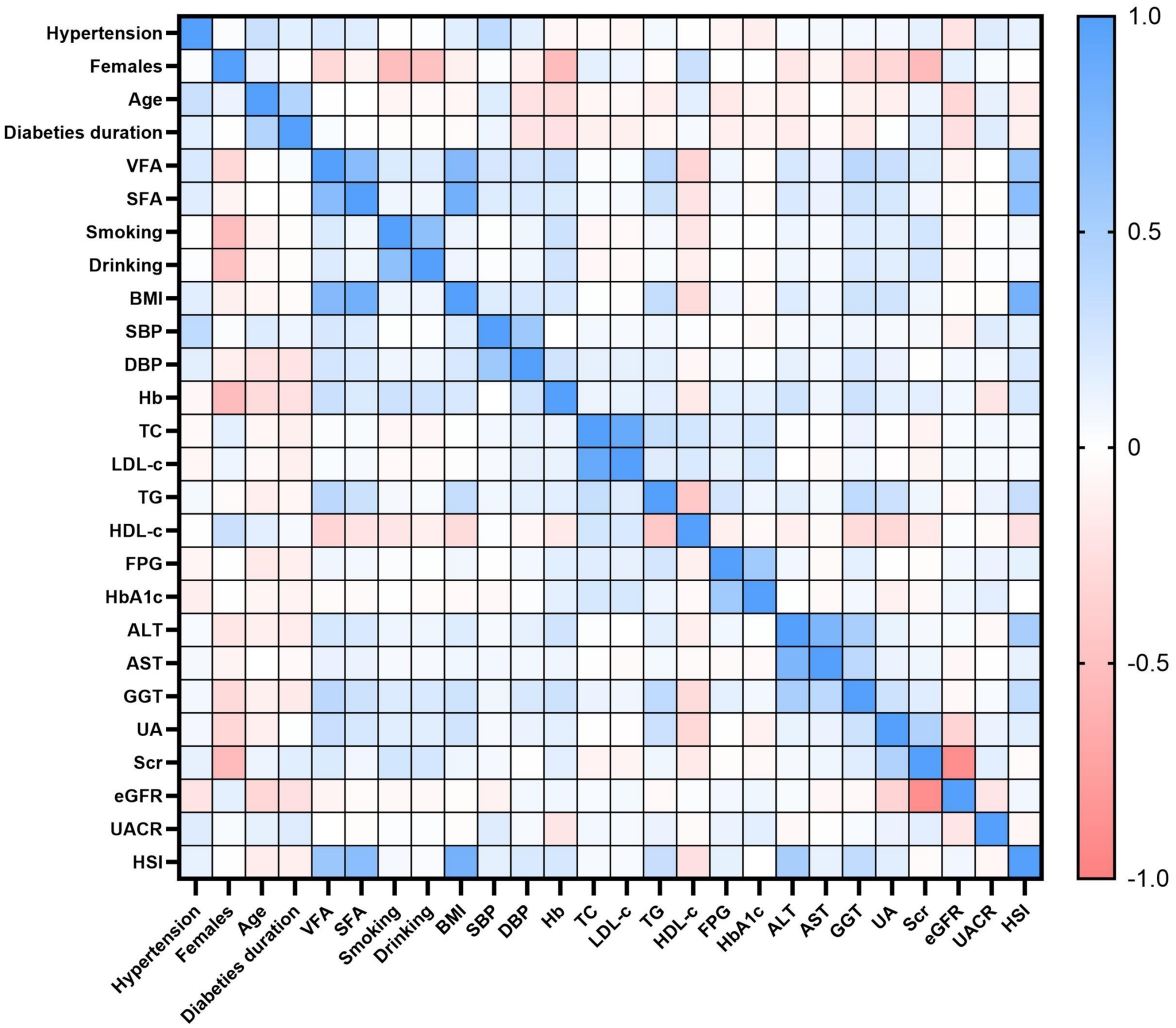
Spearman correlation heatmap among study variables. Colors denote correlation strength and direction (blue = positive; red = negative; white = near zero), with coefficients ranging from −1.0 to +1.0.

The results of the univariate analysis for hypertension in patients with T2DM are presented in Table 3. Hypertension was significantly and positively correlated with age, diabetes duration, VFA, SFA, BMI, SBP, DBP, TG, AST, GGT, UA, Scr, UACR, and HSI (all *p* < 0.05). In contrast, Hb, TC, LDL-c, FPG, HbA1c, and eGFR were negatively correlated with hypertension (all *p* < 0.05). No significant associations were found with sex, smoking status, alcohol consumption, HDL-c, or ALT (all *p* > 0.05). Figure 2 displays a heatmap of the Spearman correlation coefficients between all study variables, ranging from −1.0 to 1.0. Blue represents a strong positive correlation (values close to +1), red indicates a strong negative correlation (values close to −1), and white shows a weak or no correlation (values close to 0).

**Table 3:** Correlation of hypertension by univariate analysis.

Variables	Correlation coefficient	*P*
Females	0.024	0.316
Age	0.316	<0.001
Diabetes duration	0.167	<0.001
VFA	0.212	<0.001
SFA	0.182	<0.001
Smoking	0.002	0.927
Alcohol consumption	0.017	0.466
BMI	0.175	<0.001
SBP	0.073	<0.001
DBP	0.159	<0.001
Hb	−0.071	0.003
TC	−0.048	0.043
LDL-c	−0.076	0.002
TG	0.062	0.010
HDL-c	0.001	0.972
FPG	−0.080	0.001
HbA1c	−0.136	<0.001
ALT	0.043	0.074
AST	0.057	0.016
GGT	0.066	0.006
UA	0.064	0.008
Scr	0.137	<0.001
eGFR	−0.217	<0.001
UACR	0.191	<0.001
HSI	0.134	<0.001

VFA, visceral fat area; SFA, subcutaneous fat area; BMI, body mass index; SBP, systolic blood pressure; DBP, diastolic blood pressure; Hb, hemoglobin; TC, total cholesterol; LDL-c, low-density lipoprotein cholesterol; TG, triglyceride; HDL-c, high-density lipoprotein cholesterol; HbA1c, glycated hemoglobin; ALT, alanine aminotransferase; AST, aspartate aminotransferase; GGT, γ-glutamyl transpeptidase; UA, uric acid; Scr, serum creatinine; eGFR, estimated glomerular filtration rate; UACR, urinary albumin-to-creatinine ratio; HSI, hepatic steatosis index. Spearman correlation analysis was used to determine the correlation coefficients between hypertension and the listed variables.

### Logistic regression analysis

Using hypertension status (yes = 1, no = 0) as the dependent variable, all variables that were significant in the univariate analysis were entered into a multivariate logistic regression model (enter method).

As shown in Table 4, three variables—HSI (OR = 1.054, 95% CI: 1.025–1.085, *p* < 0.001), VFA (OR = 1.009, 95% CI: 1.006–1.012, *p* < 0.001), and age (OR = 1.066, 95% CI: 1.054–1.079, *p* < 0.001)—were identified as independent risk factors for hypertension after adjustment for BMI, smoking, and other potential confounders.

**Table 4:** Independent factors associated with hypertension by logistic regression analysis.

Variables	B	SE	Wals	*P*	OR	95.0% CI for OR
HSI	0.053	0.015	13.220	<0.001	1.054	1.025–1.085
VFA	0.009	0.002	26.069	<0.001	1.009	1.006–1.012
Age	0.064	0.006	111.935	<0.001	1.066	1.054–1.079
TC	0.199	0.102	3.782	0.052	1.221	0.998–1.492
LDL-c	−0.322	0.124	7.152	0.007	0.717	0.562–0.915
HbA1c	−0.106	0.030	12.831	<0.001	0.899	0.849–0.953
UACR	0.000	<0.001	7.867	0.005	1.000	1.000–1.000
EGFR	−0.006	0.002	9.573	0.002	0.994	0.990–0.998

HSI, hepatic steatosis index; VFA, visceral fat area; TC, total cholesterol; LDL-c, low-density lipoprotein cholesterol; HbA1c, glycated hemoglobin; UACR, urinary albumin-to-creatinine ratio; eGFR, estimated glomerular filtration rate. SE, standard error; CI, confidence interval; OR, odds ratio.

In contrast, LDL-c (OR = 0.717, 95% CI: 0.562–0.915, *p* = 0.007), HbA1c (OR = 0.899, 95% CI: 0.849–0.953, *p* < 0.001), and eGFR (OR = 0.994, 95% CI: 0.990–0.998, *p* = 0.002) were inversely associated with hypertension. Additionally, UACR showed a weak but significant positive association with hypertension (OR = 1.000, 95% CI: 1.000–1.000, *p* = 0.005), while TC demonstrated a borderline association (OR = 1.221, 95% CI: 0.998–1.492, *p* = 0.052).

## Discussion

This study demonstrated that the HSI is independently associated with hypertension in patients with T2DM, even after adjusting for major confounders such as BMI and smoking. These findings suggest that HSI may serve as a simple indicator for identifying T2DM patients at increased risk of hypertension, and future large-scale longitudinal studies will be essential to confirm these associations and clarify their clinical significance.

The relationship between HSI and hypertension may stem from shared pathophysiological mechanisms between MASLD and hypertension (15). MASLD, commonly associated with T2DM, is also strongly linked to other metabolic complications such as obesity, hypertension, and dyslipidemia (16). These conditions often coexist and interact, contributing to the development and progression of MASLD (8). Furthermore, MASLD is frequently associated with lipid abnormalities, including elevated TG and low HDL-c, which further link it to dyslipidemia and highlight its role in broader metabolic dysfunction (17). As a key component of metabolic syndrome, MASLD shares mechanisms such as insulin resistance, chronic low-grade inflammation, and endothelial dysfunction, which are critical in hypertension development (18, 19). Previous studies have demonstrated that liver fat accumulation can activate oxidative stress, induce inflammatory cytokine release, and impair endothelial function, all of which further elevate blood pressure (20, 21). Additionally, liver dysfunction has been shown to activate the renin-angiotensin-aldosterone system (RAAS), further increasing hypertension risk (22, 23).

Sex hormones play a crucial role in regulating blood pressure. Estrogen, in females, has vasodilatory effects, primarily through increasing nitric oxide production and improving endothelial function, which helps maintain vascular flexibility and lower blood pressure (24). After menopause, the decline in estrogen levels is associated with an increased risk of hypertension (25). In males, testosterone tends to increase vascular resistance, promote sodium retention, and enhance sympathetic nervous system activity, which may contribute to elevated blood pressure (26). Additionally, androgen excess in both sexes can lead to metabolic dysfunction, including insulin resistance, dyslipidemia, and elevated blood pressure (26).

Unlike imaging-based methods, HSI reflects not only hepatic steatosis but also integrates several clinical parameters linked to hypertension. Elevated ALT and AST indicate hepatic injury and metabolic stress, both of which contribute to vascular dysfunction (27, 28). BMI, as a measure of adiposity, is strongly associated with insulin resistance, sympathetic activation, and increased cardiovascular load, all of which elevate blood pressure (29, 30). Importantly, because the HSI scoring system incorporates diabetes status as an additional component, its application in T2DM patients may provide an even more accurate reflection of their cumulative metabolic burden. By combining these factors, HSI captures multiple biological pathways relevant to blood pressure regulation, which may explain its strong association with hypertension in T2DM patients observed in our study. Furthermore, beyond hypertension, HSI has shown potential for diagnosing MASLD and may be linked to liver cancer risk, particularly in diabetic patients or those with MASLD (12). However, further research is needed to validate these associations and explore the role of HSI in assessing liver cancer risk.

In addition to HSI, our study identified VFA and age as significant risk factors for hypertension in T2DM patients, consistent with prior evidence linking central adiposity and aging to elevated blood pressure (31). Increased VFA reflects greater visceral adiposity, which promotes insulin resistance, dysregulated adipokine secretion, and systemic inflammation, thereby impairing blood pressure regulation (32). In T2DM patients, excessive visceral fat not only contributes to hypertension but also amplifies the risk of diabetes-related complications (33, 34), highlighting the importance of comprehensive metabolic assessment and targeted intervention in this population.

Furthermore, our analysis revealed that several metabolic and renal indicators—including TC, LDL-c, UACR, HbA1c, and eGFR—were independently associated with hypertension in T2DM patients. Elevated TC and LDL-c may exacerbate endothelial dysfunction and accelerate atherosclerosis, thereby increasing vascular resistance (35). Higher UACR and reduced eGFR indicate subtle renal impairment, which can contribute to sodium retention, RAAS activation, and vascular remodeling, further promoting hypertension (36). Interestingly, lower HbA1c was also associated with hypertension, which may reflect the complex interplay between age, glycemic control, and long-term diabetes management. Collectively, these findings highlight the multifactorial and systemic nature of hypertension in T2DM, and support the use of HSI as an integrative marker that captures both hepatic and broader metabolic risk, providing a practical tool for early risk stratification and targeted lifestyle or pharmacologic interventions.

## Limitations

This study employed a cross-sectional design and retrospectively analyzed a large sample of inpatient data collected over a three-year period, identifying an association between HSI and hypertension in patients with T2DM. However, due to the study design, causal relationships cannot be inferred, and long-term trends remain undetermined. Furthermore, unmeasured confounding factors, such as diet, physical activity, and medication use, may have influenced the observed associations, and these factors were not systematically controlled for. Additionally, most hypertensive participants had a prior diagnosis and may have been receiving antihypertensive therapy, which could have influenced blood pressure levels and study outcomes. Thirdly, secondary causes of hypertension were not systematically screened, leaving the possibility of residual confounding. Finally, the study population was restricted to Chinese T2DM patients, limiting the generalizability of the findings to other populations. Finally, while HSI reflects hepatic steatosis, it cannot distinguish the severity or etiology of fatty liver, which may limit its interpretability in certain clinical contexts.

## Conclusion

In conclusion, this study is the first to demonstrate a significant and independent positive association between the HSI and hypertension in T2DM patients. While these findings are associative rather than causal, they suggest that HSI could serve as a simple and practical tool for identifying individuals at higher risk of hypertension in the T2DM population. Given its potential, HSI warrants further exploration through prospective and interventional studies to validate its role as a clinical screening tool and assess its utility in broader populations.

## Data Availability

The raw data supporting the conclusions of this article will be made available by the authors, without undue reservation.
